# Co-administration of aspirin and allogeneic adipose-derived stromal cells attenuates bone loss in ovariectomized rats through the anti-inflammatory and chemotactic abilities of aspirin

**DOI:** 10.1186/s13287-015-0195-x

**Published:** 2015-10-16

**Authors:** Hao Liu, Wei Li, Yunsong Liu, Xiao Zhang, Yongsheng Zhou

**Affiliations:** The Central Laboratory, Peking University School and Hospital of Stomatology, Beijing, 100081 China; Department of Prosthodontics, Peking University School and Hospital of Stomatology, 22 Zhongguancun South Avenue, Haidian District, Beijing, 100081 China; National Engineering Lab for Digital and Material Technology of Stomatology, Peking University School and Hospital of Stomatology, Beijing, 100081 China

**Keywords:** Allogeneic adipose-derived stromal cells, Aspirin, Osteoporosis, Bone regeneration, Cell homing

## Abstract

**Introduction:**

Osteoporosis is a syndrome of excessive skeletal fragility characterized by the loss of mass and deterioration of microarchitecture in bone. Single use of aspirin or adipose-derived stromal cells (ASCs) has been recognized recently to be effective against osteoporosis. The goal of the study was to evaluate the osteogenic effects of the co-administration of aspirin and allogeneic rat adipose-derived stromal cells (rASCs) on ovariectomized (OVX)-induced bone loss in rats. The underlying mechanisms were investigated *in vitro* and *in vivo*.

**Methods:**

Firstly, allogeneic rASCs were isolated and cultured, and the conditioned medium (CM) from the maintenance of rASCs was collected. Secondly, the OVX rats were administrated CM, rASCs, aspirin (ASP) or rASCs + ASP, respectively. Twelve weeks later, the anti-inflammatory and osteogenic effects were assessed by micro-CT, undecalcified histological sections, dynamic histomorphometric analyses and serologic assays for biochemical markers. Finally, a Transwell migration assay *in vitro* and cell-trafficking analyses *in vivo* were used to explore the effects of aspirin on rASC migration.

**Results:**

Systemic administration of aspirin and rASCs attenuated OVX-induced bone loss better than single use of aspirin or ASCs (*p* < 0.05, respectively). Next, we analyzed the underlying mechanisms of the anti-inflammatory and chemotactic abilities of aspirin. Aspirin suppressed serum levels of the pro-inflammatory cytokines on tumor necrosis factor-α (TNF-α) and interferon-γ (IFN-γ), and the anti-inflammatory ability was positively associated with bone morphometry. Also, aspirin exhibited excellent chemotactic effects *in vitro* and accelerated the homing of allogeneic rASCs into bone marrow during early *in vivo* stages.

**Conclusions:**

Co-administered aspirin and allogeneic ASCs can partially reverse OVX-induced bone loss in rats. This effect appears to be mediated by the anti-inflammatory and chemotactic abilities of aspirin.

## Introduction

Osteoporosis is a syndrome of excessive skeletal fragility characterized by loss of the mass and deterioration of the microarchitecture of bone, which leads to fragility fractures [1]. The prevalence of osteoporosis has increased rapidly among the elderly as life expectancy has increased in industrialized countries [2].

Two major pharmacologic approaches to the treatment of osteoporosis are anabolic agents such as parathyroid hormone [3, 4] (which stimulate bone formation) or anti-resorptive agents such as bisphosphonates, calcitonin, raloxifene, and estrogen (which inhibit bone resorption) [5, 6]. These therapies have been shown to increase bone mineral density (BMD) and reduce the risk of fractures, but long-term safety and efficacy are ongoing concerns [7]. Thus, new safe and effective modalities are desired to ameliorate osteoporotic conditions.

Stem-cell therapy has been investigated for the repair and regeneration of damaged tissue in various conditions (e.g., myocardial ischemia [8, 9], stroke [10, 11], diabetes mellitus [12, 13], and cartilage/bone defects [14–16]) owing to the potential for multi-lineage differentiation of stem cells. Bone marrow (BM) has been most commonly used as a source of mesenchymal stem cells (MSCs), but the number and capacity for multi-lineage differentiation decline with age or health of donors [17–19]. Moreover, obtaining BM is an invasive procedure that can cause complications such as pain, bleeding, and infection.

To circumvent these limitations, adipose-derived stromal cells (ASCs) have been used recently as alternative sources of MSCs. ASCs have the following advantages over other sources of MSCs: multi-lineage MSCs have similar potential to that of bone marrow-derived mesenchymal stem cells (BMMSCs); ease of harvesting; and abundant sources [20–23]. Furthermore, by avoiding difficulties in obtaining sufficient numbers of autologous stem cells and decreased osteogenic potential of ASCs as a result of donor aging [24], ASCs from allogeneic donors could constitute a valuable alternative source of stem cells for therapeutic use because ASCs have superior immunomodulatory effects than other sources of MSCs. Taken together, ASCs may be a better source of stem cells for the treatment of osteoporosis [25, 26].

Aspirin is a widely used non-steroidal anti-inflammatory drug. Epidemiologic studies have suggested that aspirin might have a moderate beneficial effect on BMD in post-menopausal women [27]. Recently, Liu *et al.* reported that implantation of BMMSCs/hydrogel/aspirin resulted in improvement of bone generation in calvarial defects by improving the osteogenesis of, and by inhibiting the apoptosis of, BMMSCs [28]. Moreover, several lines of evidence have suggested that aspirin can inhibit osteoclastogenesis and improve osteogenesis by suppression of expression of pro-inflammatory cytokines, inhibition of activity of cyclooxygenase 1 and 2, and prostaglandin-E_2_, and up-regulation of telomerase activity in BMMSCs [29–32]. Thus, we speculated that aspirin could favor stem cell-mediated bone regeneration by affecting multiple biological pathways.

Hu *et al.* demonstrated that aspirin can induce angiogenesis by promoting migration of endothelial progenitor cells [33]. Brunelli *et al.* also reported that HCT 1026 (a nitric oxide-donating agent with nonsteroidal anti-inflammatory activity) enhanced the therapeutic efficacy of arterially delivered donor stem cells by increasing their ability to migrate and reconstitute muscle fibers [34]. In the above-mentioned studies, aspirin or other nonsteroidal anti-inflammatory agents improved angiogenesis or reconstituted muscle fibers by promoting the migration of stem (progenitor) cells to the target organ. Therefore, we hypothesized that aspirin can improve osteogenesis (at least in part) by enhancing homing of stem cells into BM.

We wished to evaluate the effects of co-administration of aspirin and allogeneic rat adipose-derived stromal cells (rASCs) on ovariectomized (OVX)-induced bone loss in rats, and investigated the underlying mechanism of these effects *in vitro* and *in vivo*.

## Methods

### Ethical approval of the study protocol

All animal experiments were approved by the Animal Care and Use Committee of Peking University Health Science Center (approval number: LA2014233; Beijing, China).

### Isolation and maintenance of rASCs and preparation of conditioned medium

All materials were purchased from Sigma–Aldrich (Saint Louis, MO, USA) unless stated otherwise. For rASCs, Lewis rats were euthanized at eight weeks of age by CO_2_ asphyxiation according to standard protocols. Visceral fat pads were minced and digested by type-I collagenase, filtered through cell strainers (pore size, 100 μm; Biologix, Lenexa, KS, USA) and centrifuged for 10 minutes at 380 × *g* at room temperature. Cells were cultivated in maintenance medium [Dulbecco’s modified Eagle’s medium (DMEM) containing 10 % fetal bovine serum (FBS), 100 U/mL penicillin G, and 100 mg/mL streptomycin] at 37 °C in an atmosphere of 5 % CO_2_. rASCs of passage 3–5 were used and all cell-based experiments were repeated at least three times.

To prepare the conditioned medium (CM) of rASCs, cells were detached upon reaching 80 % confluence in a 10-cm^2^ dish and cultured for 12 h by DMEM containing 2 % FBS. Next, rASCs were cultured by a serum-free DMEM for 12 h and the supernatant was collected and sterilized by passage through a filter (pore size, 0.22 μm).

### Animals and administration procedure

Rats were allowed free access to water and a maintenance diet in a 12-h light–dark cycle at a room temperature of 21 ± 2 °C. Rats were housed in groups of ≤5.

Eight-week-old female F344 rats (n = 70) were obtained from Vital River Inc. (Beijing, China). Bilateral OVX or sham operation was undertaken by standard methods under general anesthesia using pentobarbital sodium (50 mg/kg, i.p.).

Four weeks after surgery, rats were assigned randomly into seven groups of ten: (1) sham rats were injected with vehicle (Sham group); (2) OVX rats were injected with vehicle (OVX); (3) OVX rats were injected with zoledronate (ZOL); (4) OVX rats were injected with allogeneic rASCs CM (ASC CM); (5) OVX rats were injected with allogeneic rASCs (ASC), (6) OVX rats were given a gavage of aspirin (ASP), (7) OVX rats were injected with rASCs and given a gavage of aspirin (ASC + ASP) .

On days 1, 2, 8, and 9, 200 μL of CM (total of 800 μL; ASC CM group) or 1.5 × 10^6^ allogeneic rASCs in 200 μL of DMEM (total of 6 × 10^6^ cells; ASC and ASC + ASP groups) were injected into the lateral tail vein. Then, 100 mg/kg body weight of aspirin in 2 mL of physiologic (0.9 %) saline (1 % DMSO assisted solubilization) was given by gavage every day of the total study period in ASP and ASC + ASP groups. In sham and OVX groups, DMEM and physiologic saline were administered each time. As a positive control, zoledronate (100 μg/kg, s.c.) was administered on day-1 and day-8 in the ZOL group.

Twelve weeks later, after euthanasia, the right tibia from each animal was dissected free from soft tissue thoroughly and fixed in 10 % neutral-buffered formalin. Subsequently, it was used for micro-computed tomography (CT) and histological slicing.

### Micro-CT analyses

To evaluate the mass and microarchitecture in bone among the seven groups, micro-CT was undertaken using an Inveon MM system (Siemens, Munich, Germany). Images were acquired at an effective pixel size of 8.82 μm, voltage of 80 kV, current of 500 μA and exposure time of 1,500 ms in each of the 360 rotational steps. Parameters were calculated using an Inveon Research Workplace (Siemens) as follows: bone volume/total volume (BV/TV), bone surface area/bone volume (BS/BV), trabecular thickness (Tb.Th), trabecular number (Tb.N), trabecular separation (Tb.Sp) and BMD in the trabecular region (1–2 mm distal to the proximal epiphysis) according to guidelines set by the American Society for Bone and Mineral Research [35].

### Slicing of undecalcified bones and dynamic histomorphometric analyses

Rats were injected with alizarin-3-methyliminodiacetic acid (30 mg/kg body weight, i.p.) ten days before euthanasia and calcein (20 mg/kg body weight, i.p.) three days before. The tibia was fixed as described above and dehydrated in ethanol followed by embedding in destabilized methyl methacrylate resin. Next, sections were ground and polished to 40–60 μm by an EXAKT precision cutting and grinding system (EXAKT Apparatebau, Hamburg, Germany) and stained (hemotoxylin and eosin). Dynamic histomorphometry was undertaken using Bioquant software (BioQuant, San Diego, CA, USA).

### Assay for biochemical markers

For detection of biochemical markers of bone turnover and pro-inflammatory cytokines, blood samples were acquired from the aorta ventralis upon euthanasia. Serum was separated and stored at −80 °C. Serum levels of procollagen 1 N-terminal peptide (P1NP), bone alkaline phosphatase (ALP), and tartrate-resistant acid phosphatase (TRAP) were measured using the relevant enzyme-linked immunoassay (ELISA) kits (IDS, Frankfurt, Germany). Serum levels of calcium were measured using a Plasma Emission Spectrometer (iCAP 6000; Thermo Fisher Scientific, Waltham, MA, USA). Serum levels of tumor necrosis factor (TNF)-α and interferon (IFN)-γ were measured using the relevant ELISA kits (eBioscience, San Diego, CA, USA). Samples were measured in duplicate (at least).

### Cell-migration assay *in vitro* and *in vivo*

To explore the effects of aspirin on migration of rASCs, a transwell migration assay in *vitro* and cell-trafficking analyses *in vivo* were used.

For the transwell migration assay, 1 × 10^5^ cells from passage 4 were loaded into the upper chamber of a 24-well transwell plate (pore size, 8 μm; Corning, Corning, NY, USA) and 600 μL DMEM containing different concentrations of aspirin added to the lower chamber. Twenty-four hours later, cells on the upper surface of the membranes were removed with a cotton swab. Cells that had migrated to the lower surface of the membrane were fixed with 10 % neutral-buffered formalin and stained for 15 min with 0.5 % crystal violet. The number of cells that had migrated into the lower chamber was counted twice in five randomly selected microscopic fields (at × 400 magnification) per filter by blind evaluations by two independent assessors. All aspirin concentrations were repeated at least three times.

For cell-trafficking analyses, a parallel assay using fluorescent dye-labeled cell injection was used. Forty 12-week-old F344 rats were divided into two groups of twenty animals four weeks after surgery: (1) OVX rats were given a gavage of vehicle (OVX-Vehicle); and (2) OVX rats were given a gavage of aspirin (OVX-ASP). A total of 100 mg/kg aspirin in 2 mL of physiologic saline was given by gavage on each day of the total study period. After one week of gavage, allogeneic rASCs were labeled with 5 μM CM-DiL fluorescent dye (CellTracker™ CM-DiL; Invitrogen, Carlsbad, CA, USA) according to the manufacturer’s instructions for adherent cells. A total of 1.5 × 10^6^ CM-DIL-labeled allogeneic rASCs from Lewis rats in 200 μL of DMEM were injected into F344 rats after four weeks of OVX (n = 4, at each time point of then group). Rats were euthanized at 6, 12, 24, 48, and 72 h after cell injection. BM cells from the femur were obtained as described previously [36] and analyzed for CM-DiL-positive cells (10^4^ cells per sample) with LSRFortessa™ (BD Biosciences, Franklin Lakes, NJ, USA).

### Statistical analysis

Data are shown as the mean ± standard deviation. SPSS v16.0 (IBM, Armonk, NY, USA) was used for statistical analyses. Independent two-tailed Student’s *t*-test or one-way analysis of variance (ANOVA) with post hoc least significant difference (LSD) were performed, and p-values less than 0.05 were considered significant.

## Results

### Radiologic and histologic evaluation of the mass and microarchitecture of bone

**Fig. 1:**
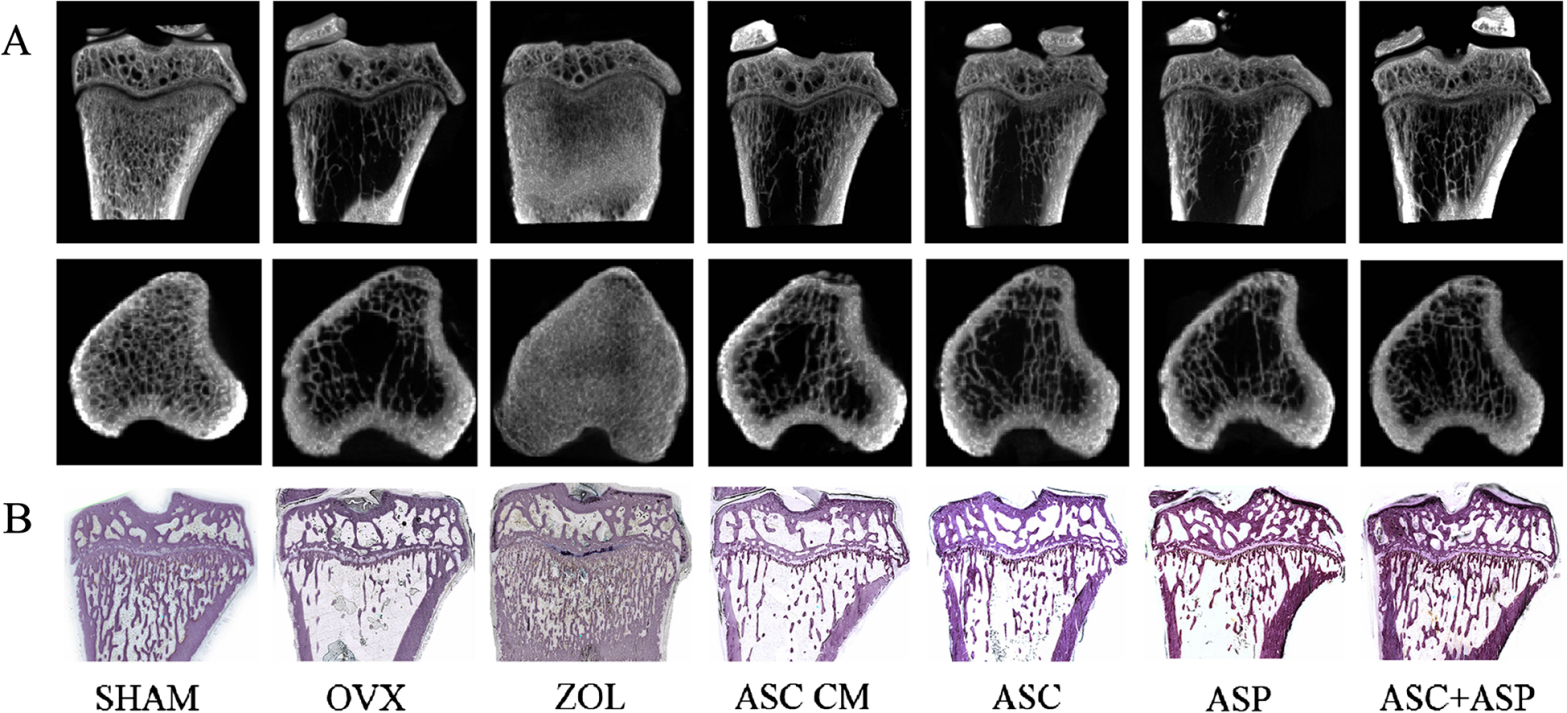
Representative images of changes in bone microarchitecture in rats. Tibias of F344 rats were dissected and evaluated ex vivo by micro-CT (**a**) and slicing of undecalcified tissues (H & E staining); (**b**) eight weeks after treatment

To assess the mass and microarchitecture of bone, BMD and bone morphometry were carried out using micro-CT (Fig. 1a) and the results are shown in Table 1. For BMD, micro-CT analyses of the proximal tibia *ex vivo* demonstrated that the OVX groups (OVX, ASC CM, ASC, ASP, and ASC + ASP groups, n = 50) exhibited significantly less BMD compared with the sham group except the ZOL group (*p* < 0.001). Interestingly, the BMD of the ASC CM group increased significantly compared with the OVX and ASC groups (OVX group *p* = 0.012, ASC group *p* = 0.032). However, inconsistent with BMD results, the ASC CM group showed a poor effect upon bone microarchitecture among the treatment groups (ASC CM, ASC, ASP, and ASC + ASP groups). The ASC CM group had a dramatically decreased BV/TV compared to the ASC + ASP group (*p* = 0.001) and a dramatically decreased Tb.Th compared with the other treatment groups (ASC group *p* = 0.005, ASP group *p* = 0.033, ASC + ASP group *p* < 0.001). The ASC CM group had significantly increased BS/BV compared with the other treatment groups (ASC group, *p* = 0.009; ASP group, *p* = 0.045; ASC + ASP group, *p* < 0.001). The ASC + ASP group had a significantly increased BV/TV, Tb.N, and Tb.Sp compared with the ASC group and ASP group (for the ASC group and ASP group, respectively, BV/TV: *p* = 0.006 and *p* = 0.002; Tb.N: *p* = 0.006 and *p* = 0.004; Tb.Sp: *p* = 0.020 and *p* = 0.013).

**Table 1:** Mineral density and histomorphometry of tibias in the seven groups of rats

Groups	Sham	OVX	ZOL	ASC CM	ASC	ASP	ASC + ASP
BMD (g/cm^3^)	1.791 ± 0.048	1.445 ± 0.031 ^a^	1.969 ± 0.069 ^a,b^	1.497 ± 0.031 ^a,b,c^	1.457 ± 0.045 ^a,c,d^	1.466 ± 0.034 ^a,c^	1.470 ± 0.024 ^a,c^
BV/TV (%)	44.009 ± 2.432	23.676 ± 2.848 ^a^	87.026 ± 2.388 ^a,b^	29.216 ± 2.435 ^a,b,c^	30.202 ± 4.569 ^a,b,c^	29.378 ± 3.394 ^a,b,c^	34.521 ± 1.965 ^a,b,c,d,e,f^
BS/BV (mm^−1^)	33.857 ± 2.247	37.474 ± 1.611^a^	12.362 ± 2.142 ^a,b^	35.788 ± 1.108 ^c^	33.214 ± 2.372 ^b,c,d^	33.784 ± 2.210 ^b,c,d^	32.162 ± 1.642 ^b,c,d^
Tb.Th (mm)	0.059 ± 0.003	0.053 ± 0.002 ^a^	0.156 ± 0.003 ^a,b^	0.056 ± 0.002 ^a,c^	0.060 ± 0.004 ^b,c,d^	0.059 ± 0.004 ^b,c,d^	0.062 ± 0.003 ^b,c,d^
Tb.N (mm^−1^)	7.376 ± 0.204	4.427 ± 0.473 ^a^	8.008 ± 0.252 ^a,b^	5.223 ± 0.400 ^a,b,c^	4.979 ± 0.540 ^a,b,c^	4.934 ± 0.359 ^a,b,c^	5.551 ± 0.410 ^a,b,c,e,f^
Tb.Sp (mm)	0.077 ± 0.008	0.174 ± 0.022 ^a^	0.026 ± 0.007 ^a,b^	0.137 ± 0.015 ^a,b,c^	0.143 ± 0.025 ^a,b,c^	0.144 ± 0.018 ^a,b,c^	0.119 ± 0.012 ^a,b,c,e,f^

Parameters of bone mineral density (BMD) and microarchitecture (BV/TV, BS/BV, Tb.Th, Tb.N, Tb.Sp) were measured in the trabecular bone of the proximal tibia (1–2 mm distal to the proximal physis) using micro-CT. Data are the mean ± SD, n = 70

*OVX* ovariectomized, *ZOL* zoledronate, *ASC* adipose-derived stromal cells, *CM* conditioned medium, *ASP* aspirin, *BV/TV* bone volume/total volume, *BS/BV* bone surface area/bone volume, *Tb.Th* trabecular thickness, *Tb.N* trabecular number, *Tb.Sp* trabecular separation

^a^ *p* < 0.05 *vs*. Sham, ^b^ *p* < 0.05 *vs*. OVX, ^c^ *p* < 0.05 *vs*. ZOL, ^d^ *p* < 0.05 *vs*. ASC CM, ^e^ *p* < 0.05 *vs*. ASC, ^f^ *p* < 0.05 *vs*. ASP

To confirm radiologic evaluation of tibias, analyses of slices of hard tissues were undertaken and the results of bone morphometry from histology were similar to those of the micro-CT (data not shown) (Fig. 1b).

### Dynamic histomorphometric analyses

**Fig. 2:**
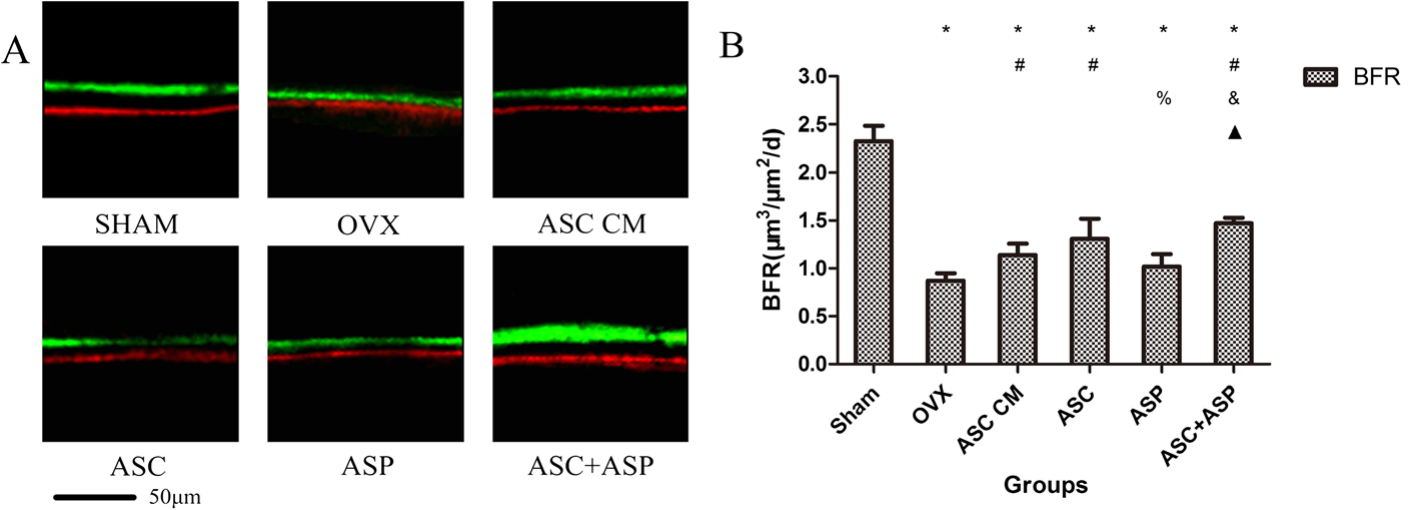
Dynamic histomorphometric analyses of rat tibias. **a** Representative fluorescence images obtained from tibias after double labeling with alizarin red and calcein. Scale bar = 50 μm. **b** Bone formation rate (BFR) was measured from the tibia using Bio-quant software. Data are the mean ± SD. *p < 0.05 vs. Sham, #*p* < 0.05 vs. OVX, &*p* < 0.05 vs. ASC CM, %*p* < 0.05 vs. ASC, black triangle *p* < 0.05 vs. ASP. *OVX* ovariectomized, *ASC* adipose-derived stromal cells, *ASP* aspirin

Consistent with the analyses of bone microarchitecture, dynamic histomorphometric analyses also revealed significant increases in the bone formation rate (BFR) in the ASC + ASP group compared with the OVX, ASC CM, and ASP groups (OVX group, *p* < 0.001; ASP group, *p* < 0.001; ASC CM group, *p* = 0.006) (Fig. 2). However, the BFR of the ASP group was reduced significantly compared with the ASC group (*p* = 0.006). The BFR of the OVX groups (OVX, ASC CM, ASC, ASP, and ASC + ASP groups, n = 50) was decreased significantly compared with the sham group (*p* < 0.001). The ZOL group showed irregular fluorescence and calculation of the BFR was difficult because osteoclast-directed bone resorption (which is usually coupled with bone formation) was inhibited (data not shown).

### Serological assessment of the formation and resorption of bone

**Fig. 3:**
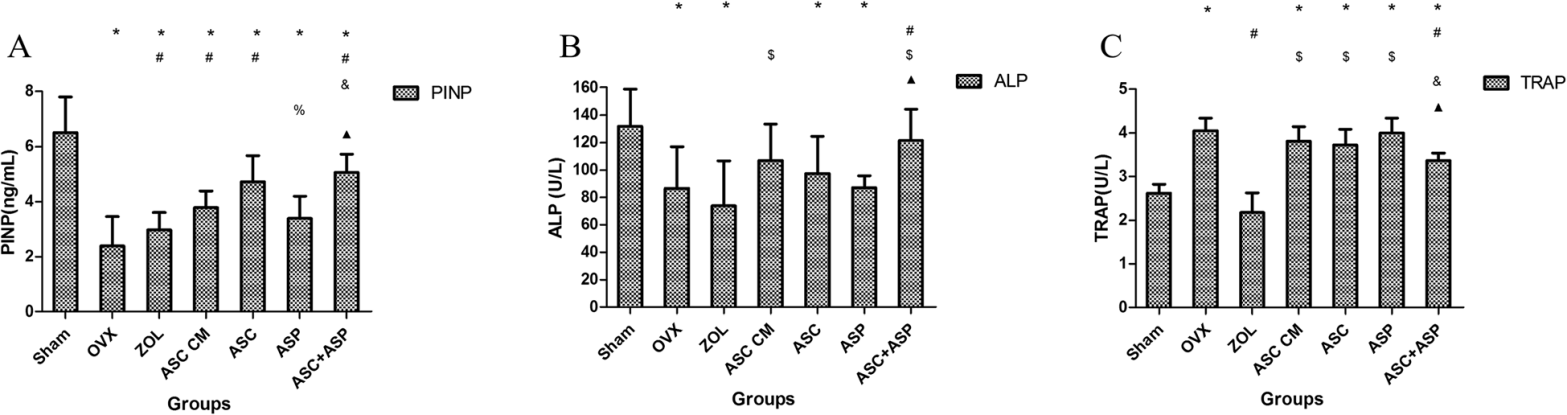
Serum levels of biochemical markers of bone turnover. Serum levels of P1NP (**a**) and ALP (**b**) (marker of bone formation), and TRAP 5b (marker of bone resorption) (**c**) eight weeks after treatment. Data are the mean ± SD of experiments carried out in triplicate. *p < 0.05 vs. Sham, #*p* < 0.05 vs. OVX, $*p* < 0.05 vs. ZOL, &*p* < 0.05 vs. ASC CM, %*p* < 0.05 vs. ASC, black triangle *p* < 0.05 vs. ASP. *P1NP*, procollagen 1 N-terminal peptide, *ALP* alkaline phosphatase, *TRAP*, tartrate-resistant acid phosphatase, *OVX* ovariectomized, *ZOL* zoledronate, *ASC* adipose-derived stromal cells, *ASP* aspirin

Data from the ELISA of P1NP, ALP, and TRAP from each group are shown in Fig. 3. As expected, serum levels of P1NP from the OVX groups (OVX, ZOL, ASC CM, ASC, ASP, and ASC + ASP groups, n = 60), a marker of bone formation, were significantly lower than those of the sham group (OVX, ZOL, ASC CM, and ASP groups, *p* < 0.001; ASC group, *p* = 0.001; ASC + ASP group, *p* = 0.011). In accordance with bone histomorphometric analyses, serum levels of P1NP were dramatically higher in the ASC + ASP group than in OVX, ASC CM, and ASP groups (OVX group, *p* < 0.001; ASC CM group, *p* = 0.027; ASP group, *p* = 0.007). Also, P1NP levels in the ASP group were decreased significantly compared with those of the ASC group (*p* =0.022) (Fig. 3a).

Similar to the data from the ELISA for P1NP, serum levels of ALP from the OVX, ZOL, ASC, and ASP groups (n = 40) were significantly lower than those of the sham group (OVX group, *p* = 0.002; ZOL group, *p* < 0.001; ASC group, *p* = 0.018; and ASP group, *p* = 0.003). Moreover, ALP levels in the ASC + ASP group dramatically increased compared with the OVX, ZOL, and ASP groups (OVX group, *p* = 0.016; ZOL group, *p* = 0.001; ASP group, *p* = 0.018) (Fig. 3b).

Moreover, serum levels of TRAP (a marker of bone resorption) were significantly lower in the sham group than in the OVX, ASC CM, ASC, ASP, and ASC + ASP groups (OVX, ASC CM, ASC and ASP groups, *p* < 0.001; ASC + ASP group, *p* = 0.001). Notably, owing to the anti-resorption effect of zoledronate in bone, TRAP levels in the ZOL group were decreased significantly compared with the OVX, ASC CM, ASC, and ASP groups (OVX, ASC CM, and ASP groups, *p* < 0.001; ASC group, *p* = 0.001). Serum levels of TRAP in the ASC + ASP group were reduced significantly compared with those in the OVX, ASC CM, and ASP groups (OVX group, *p* = 0.004; ASC CM group, *p* = 0.038; ASP group, *p* = 0.003). However, there was no significant difference among the OVX, ASC CM, ASC, and ASP groups (Fig. 3c). These findings suggest that systemic administration of rASCs, ASC CM, or aspirin primarily affected bone formation and osteoblastic activity, and was also associated with bone resorption (especially in the ASC + ASP group).

### Serological assessment of calcium

**Fig. 4:**
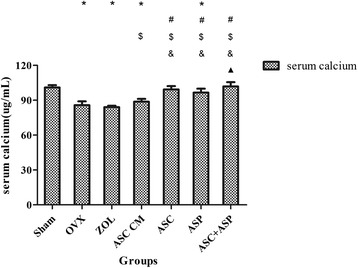
Serum levels of calcium eight weeks after treatment. Data are the mean ± SD.*p < 0.05 vs. Sham, #*p* < 0.05 vs. OVX, $*p* < 0.05 vs. ZOL, &*p* < 0.05 vs. ASC CM, black triangle *p* < 0.05 vs. ASP. *ALP* alkaline phosphatase, *OVX* ovariectomized, *ZOL* zoledronate, *ASC CM* Adipose-derived stromal cells conditioned medium, *ASP* aspirin

ELISAs suggested that serum levels of calcium in the OVX, ZOL, ASC CM, and ASP groups (OVX, ZOL, ASC CM groups, *p* < 0.001; ASP group, *p* = 0.003) decreased significantly compared with those in the sham group (Fig. 4). Calcium levels in the serum of rats in the ASC + ASP and ASC groups were comparable with those of the sham group. Serum levels of calcium in the ASC + ASP group increased significantly compared with those in the OVX, ZOL, ASC CM, and ASP groups (OVX, ZOL, and ASC CM groups, *p* < 0.001; ASP group, *p* = 0.002). However, serum levels of calcium decreased significantly in the ASC CM group compared with those in the other treatment groups (ASC, ASP, and ASC + ASP groups) (*p* < 0.001, respectively).

### Serological assessment of pro-inflammatory cytokines (TNF-α and IFN-γ)

**Fig. 5:**
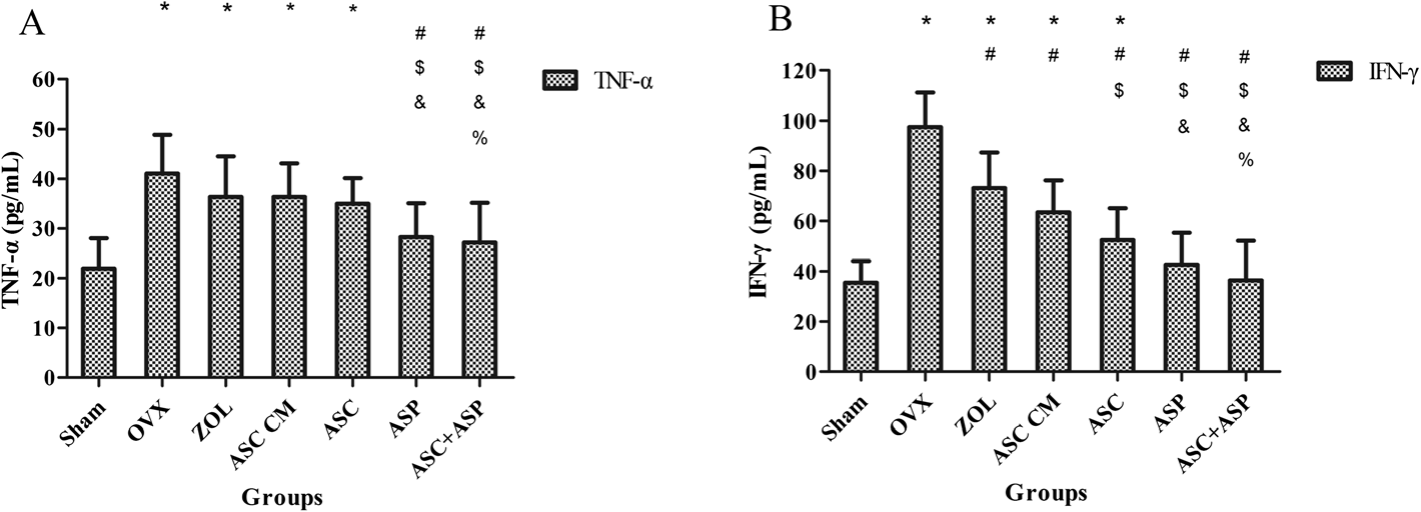
Serum levels of TNF-α and IFN-γ by ELISA eight weeks after treatment. Serum levels of TNF-α (**a**) and IFN-γ (**b**) among the seven groups. Data are the mean ± SD of experiments undertaken in triplicate. *p < 0.05 vs. Sham, #*p* < 0.05 vs. OVX, $*p* < 0.05 vs. ZOL, &*p* < 0.05 vs. ASC CM, %*p* < 0.05 vs. ASC. *OVX* ovariectomized, *ZOL* zoledronate, *ASC CM* adipose-derived stromal cells conditioned medium

Serum levels of TNF-α and IFN-γ among the seven groups are shown in Fig. 5. As expected, serum levels of TNF-α and IFN-γ were increased significantly in the OVX, ZOL, ASC CM, and ASC groups (TNF-α: OVX and ASC CM groups, *p* < 0.001; ZOL group, *p* = 0.001; ASC group, *p* = 0.002; IFN-γ: OVX and ZOL groups, *p* < 0.001; ASC CM group, *p* = 0.001; ASC group *p* = 0.036) compared with the sham group. Consistent with the results of bone microarchitecture, serum levels of TNF-α and IFN-γ were decreased significantly in the ASC + ASP group compared with the OVX, ZOL, ASC CM, and ASC groups (TNF-α: OVX group, *p* = 0.001; ZOL group, *p* = 0.020; ASC CM group, *p* = 0.012; ASC group, *p* = 0.038; IFN-γ: OVX and ZOL groups, *p* < 0.001; ASC CM group, *p* = 0.001; ASC group, *p* = 0.030). Moreover, serum levels of TNF-α and IFN-γ in the ASP group were significantly lower than those in the OVX, ZOL, and ASC CM groups (TNF-α: OVX group, *p* = 0.003; ZOL group, *p* = 0.045; ASC CM group, *p* = 0.032; IFN-γ: OVX group, *p* < 0.001; ZOL group, *p* = 0.001; ASC CM group, *p* = 0.014).

### Effect of aspirin on chemotactic ability in *vitro*

**Fig. 6:**
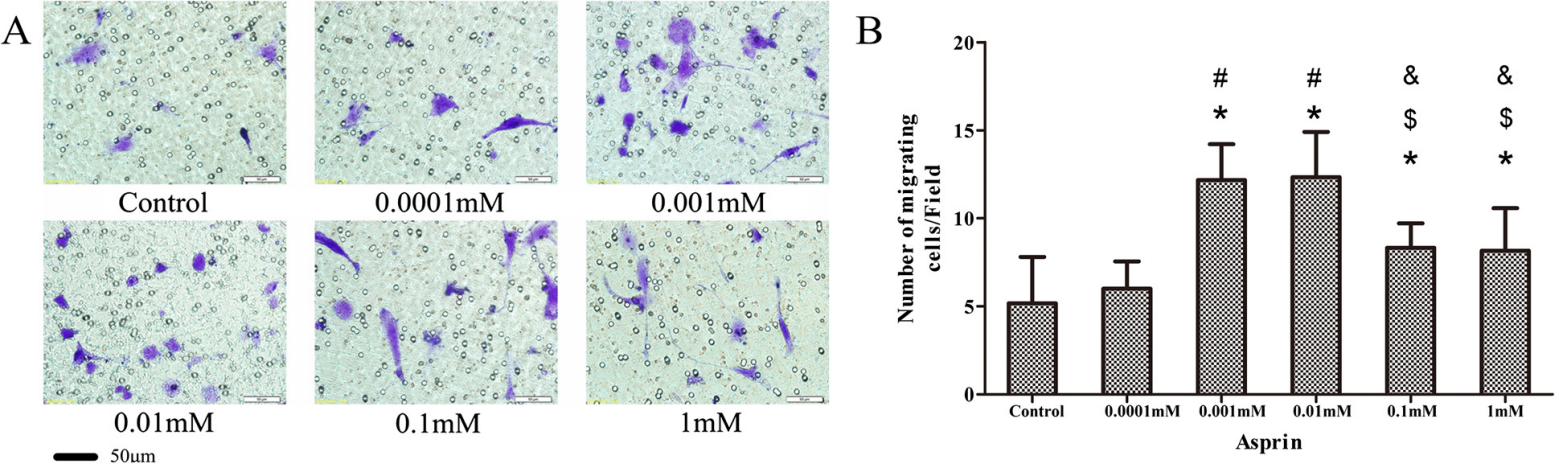
The in *vitro* chemotactic ability of aspirin by transwell assays. **a** Representative images of ASC migration at different concentrations of aspirin. Scale bar = 50 μm. **b** The number of migrating cells at different concentrations of aspirin. Data are the mean ± SD of experiments undertaken in triplicate. *p < 0.05 vs. control, #*p* < 0.05 vs. 0.0001 mM, $*p* < 0.05 vs. 0.001 mM, &*p* < 0.05 vs. 0.01 mM. *ASC* adipose-derived stromal cells

The effect of aspirin on chemotactic ability *in vitro* is shown in Fig. 6. The ability of rASCs to migrate at aspirin concentrations of 0.001, 0.01, 0.1, and 1 mM was enhanced significantly compared with that in the control group (0.001 mM and 0.01 mM, *p* < 0.001; 0.1 mM, *p* = 0.016; 1 mM, *p* = 0.022). At aspirin concentrations of 0.001 mM and 0.01 mM, cell migration was 135 % and 137 % higher than that of the control group. Moreover, significant increases were observed in the 0.001-mM and 0.01-mM groups compared with 0.1-mM (0.001 mM, *p* = 0.004; 0.01 mM, *p* = 0.003) and 1-mM groups (0.001 mM, *p* = 0.003; 0.01 mM, *p* = 0.002). However, the cell-migration ratio in the 0.0001-mM group was not increased significantly compared with that in the control group (*p* > 0.05). A significant difference between 0.1-mM and 1-mM groups was not observed.

### Effect of aspirin on homing of rASCs *in vivo*

**Fig. 7:**
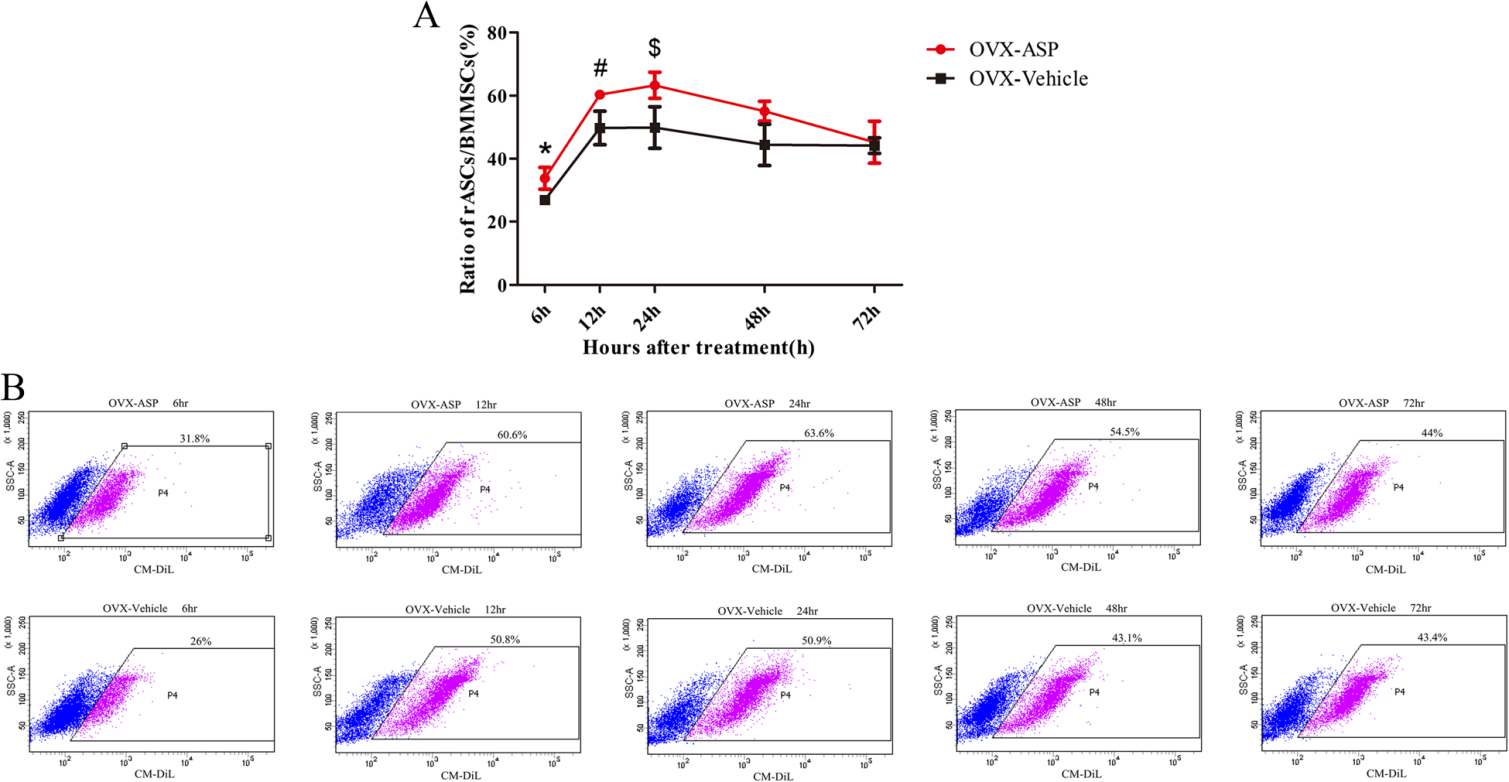
Effect of aspirin on homing of rASCs by cell-trafficking assays *in vivo*. BM cells were flushed 6, 12, 24, 48, and 72 h after injection of CM-DiL-labeled cells in the lateral tail vein and primary culture carried out. Thereafter, numbers of CM-DiL-positive cells were measured by flow cytometry. The percentage of rASCs/BMSCs in the BM at different time points (**a**) and representative images of flow cytometry (**b**) are shown. Data are the mean ± SD. *p < 0.05 vs. OVX-vehicle at 6 h, #*p* < 0.05 vs. OVX-vehicle at 12 h, $*p* < 0.05 vs. OVX-vehicle at 24 h. *rASCs* rat adipose-derived stromal cells, *BM* bone marrow, *CM* conditioned medium, *BMSCs* bone marrow stromal cells, *OVX* ovariectomized.

The effect of aspirin on homing of rASCs by cell-trafficking assays *in vivo* is shown as a curve of the ratio of rASCs/bone marrow stromal cells (BMSCs) in the BM at different time points in Fig. 7. At 6, 12, and 24 h after treatment, the ratio of rASCs/BMSCs in the OVX-ASP group was significantly greater than that in the OVX-Vehicle group (33.77 ± 3.49 % *vs.* 26.9 ± 1.56 % at 6 h, *p* = 0.036; 60.37 % ± 0.78 % *vs.* 49.7 % ± 5.32 % at 12 h, *p* = 0.027; 63.27 % ± 4.11 % *vs.* 49.9 % ± 6.58 % at 24 h, *p* = 0.041). The difference in the ratio of homing of rASCs increased gradually from 6 h to 24 h between the OVX-ASP group and OVX-Vehicle group, and reached a maximum at 24 h. At 48 h and 72 h, the ratio of rASCs/BMSCs in the OVX-ASP group was higher than that in the OVX-Vehicle group, but this difference was not significant. In the OVX-ASP group, the ratio of rASCs/BMSCs increased rapidly before 24 h after treatment and gradually declined until 72 h.

## Discussion

In the present study, we demonstrated for the first time that the co-administration of aspirin and allogeneic rASCs partially prevented OVX-induced bone loss in OVX rats. We also confirmed that the effects of co-administered aspirin and allogeneic rASCs can attenuate bone loss more effectively than either one alone. Nevertheless, the single administration of aspirin or allogeneic rASCs (and even rASCs CM) also partially prevented bone loss in comparison with the OVX control group. To fully explore the underlying mechanism of this co-operation, a combination of *in vitro* and in *vivo* experiments was carried out.

We found that aspirin decreased serum levels of the pro-inflammatory cytokines TNF-α and IFN-γ markedly. Also, aspirin enhanced the chemotactic ability of rASCs in *vitro* and accelerated homing of rASCs into BM *in vivo*. These results suggest that aspirin has comprehensive anti-inflammatory effects and promotes homing of rASCs, which might be associated with attenuating OVX-induced bone loss.

### Anti-inflammatory effect of aspirin for bone regeneration

We found that aspirin significantly suppressed serum levels of the pro-inflammatory cytokines TNF-α and IFN-γ, which is in accordance with the findings of previously reported studies [28, 37]. Meanwhile, the aspirin groups (ASP and ASC + ASP groups) showed partially prevented bone loss in OVX rats. Hence, we speculated that TNF-α and IFN-γ may play a role in bone metabolism.

Firstly, several recent lines of evidence have supported the hypothesis that IFN-γ or TNF-α accelerated osteoclastogenesis through T-cell immunity [38–41]. Studies have shown that TNF-α (which is derived from activated T cells as a result of OVX) augments macrophage colony-stimulating factor (M-CSF)-induced and receptor activator of nuclear factor-kappa B ligand (RANKL)-induced osteoclastogenesis via the TNF-α receptor p55 when estrogen deficiency induces bone loss [38, 39]. Cenci *et al.* discovered that IFN-γ enhances by the upregulated expression of class-II transactivator located in antigen-presenting cells (APC) and T lymphocytes in OVX mice, resulting in enhanced antigen presentation by macrophages, as well as enhanced T cell activation and lifespan [40]. In this research, the serum levels of TNF-α and IFN-γ in the OVX group were highest among all seven groups, which also supports the above-mentioned hypothesis. In addition, Gao *et al.* demonstrated that IFN-γ indirectly promotes bone resorption by multiple pathways (T cell activation and T cell secretion of RANKL and TNF-α), but blunts osteoclast formation through the direct targeting of osteoclast precursors. However, under conditions of estrogen deficiency, infection and inflammation, the net balance of these two opposing factors is biased toward bone resorption [41]. In summary, co-operation of TNF-α and IFN-γ enhances the proliferation and activation of T cells, and further augments M-CSF-induced and RANKL-induced osteoclastogenesis. Our findings have demonstrated that the levels of TRAP, a marker of bone resorption, in the ASC + ASP group significantly decreased compared with those in the OVX group, which is consistent with the results of the above-mentioned studies. However, the level of TRAP in the ASP group only reduced slightly compared with the OVX group. This finding appeared to be inconsistent with the observation that TNF-α and IFN-γ were suppressed by aspirin. We conjectured that perhaps a small amount of DMSO (1 % DMSO for aspirin solubilization) promoted osteoclast differentiation [42] in the OVX model and attenuated the inhibition of osteoclastogenesis. In addition, Zhang *et al.* [43] showed that aspirin inhibits osteoclast differentiation and bone resorption in a dose-dependent manner, whereby low-dose aspirin has a weak effect on osteoclast inhibition. In this study, we only administered low-dose aspirin to the rats, equivalent to 15.87 mg/kg/d aspirin for humans, as calculated by the relative body surface area between rats and humans [44]. Therefore, we determined that aspirin alone did not significantly decrease TRAP levels compared to the OVX groups as a probable consequence of the combination of low-dose aspirin and DMSO.

Secondly, TNF-α and IFN-γ promoted the apoptosis of BMMSCs and osteoblasts. Liu *et al.* demonstrated that TNF-α and IFN-γ synergistically enhance BMMSC apoptosis by the inhibition of the TNFR2/NFκB pathway and Fas internalization, and inhibit BMMSC-mediated bone formation [28]. More recently, Wang *et al.* showed that TNF-α and IFN-γ synergistically downregulate expression of FAS ligand in osteoblast progenitors and reduce the ability to induce osteoclast apoptosis by osteoblasts [45].

Taken together, the above-described studies and the results from the present study suggest that aspirin suppressed levels of pro-inflammatory cytokines, such as TNF-α and IFN-γ, and partially prevented bone loss, which may be associated with decreasing osteoclastogenesis and inhibiting the apoptosis of BMMSCs and osteoblasts. The mechanisms involved in aspirin and bone loss will be the subject of future studies.

### Chemotactic effects of aspirin on bone regeneration *in vitro* and *in vivo*

The original intention of the present study was to use aspirin as an anti-inflammatory agent to attenuate bone loss in OVX rats. Interestingly, we found that aspirin (0.001 mM–1 mM) increased the migration of rASCs *in vitro*. Reports of the effects of aspirin on the migration and homing of adipose-derived stem cells are lacking. Hu *et al.* reported that low-dose aspirin promoted the migration and adhesion of endothelial progenitor cells due to the vasculoprotective benefits of aspirin [33]. The results of the present study are consistent with those studies. However, Chen *et al.* hypothesized that aspirin decreases the proliferative, migratory, and adhesive effects of endothelial progenitor cells at higher concentrations (1 mM–10 mM) [46]. These divergent views might have resulted from the aspirin dose, variations in type and/or species of cells, as well as the *in vitro* or *in vivo* assays we employed.

Also, Brunelli *et al.* reported that HCT 1026 (an agent with nonsteroidal anti-inflammatory activity) significantly enhanced the engrafting to muscle of mesoangioblasts, a type of stem cell shown to be effective in correcting genetic defects of muscle. Interestingly, the increased homing of mesoangioblasts to muscle has been shown to be accompanied by a concomitant reduction of their number in the relevant filter organs (liver, lung, kidney, and spleen) [34]. These findings regarding enhanced stem cell homing by a nonsteroidal anti-inflammatory agent *in vivo* are similar to our current results, although the underlying mechanisms have not yet been explained. Although we have not provided histological evidence as to why aspirin promoted homing to the BM rather than elsewhere, the *in vivo* and *in vitro* cell migration induced by aspirin detected by both FCM and the transwell assays validated this finding. It may be that the ability of aspirin to induce cell migration is not specific for bone, and other organs or tissues may also benefit. However, the bone will indeed benefit if the aspirin induces rASCs to home to bone. In future studies, we will use more specific methods to investigate the mechanism of stem cell homing.

### Osteogenesis upon co-administration of aspirin and allogeneic ASCs in OVX rats

The single administration of aspirin, allogeneic rASCs or rASCs CM resulted in partial osteogenesis. However, co-administration of aspirin and allogeneic ASCs had better effects upon bone regeneration. With regard to the co-administration effects of aspirin and rASCs, aspirin improved the osteogenic microenvironment of rASCs by suppressing expression of pro-inflammatory cytokines and accelerating homing of rASCs, but allogeneic rASCs constitute an abundant source of stem cells for therapeutic use and have excellent immunosuppressive and immunomodulatory properties [47, 48]. In accordance with our conclusions, Liu Y *et al.* reported that implantation of BMMSCs/hydrogel/aspirin resulted in improvement of bone generation in calvarial defects [28]. Taken together, the co-administration of aspirin and stem cells appears to be a feasible strategy to promote stem cell-mediated bone regeneration. However, young rASCs were administered in this research, which have better osteogenic effects than aged rASCs [49]. Moreover, the more long-term osteogenic effects of young rASCs were not studied. Next, we will study the long-term osteogenic effects of administering young rASCs. Recently, Yan *et al.* [50] reported that 5-azacytidine improves the osteogenic differentiation potential of aged ASCs by DNA demethylation, which may indicate a useful area of further investigation.

Interestingly, we observed that the CM of allogeneic rASCs also partially prevented OVX-induced bone loss in rats and resulted in a higher BMD than that in the ASC + ASP group. In support of our hypothesis, Mirsaidi *et al.* showed that the osteogenic potential of human BMSCs cultured with homogeneous ASC-CM dramatically increased compared with that of BMSCs cultured with BMSC-CM [51]. Sun *et al*. discovered that *in vitro*, treatment with ASC-CM enhanced osteogenic differentiation in stromal cells and pre-osteoblasts [52]. Jee *et al.* demonstrated that the CM of human umbilical cord blood-derived mesenchymal stem cells preserved BMD and improved trabecular parameters comparable with that of the OVX group and appeared to be mediated by a paracrine mechanism rather than direct engraftment of MSCs [6]. Moreover, Banas *et al*. reported that MSCs from adipose tissue produced significantly more immunosuppressive IL-1RA than BMMSCs; also IL-1RA was a robust anti-inflammatory cytokine and inhibited the binding of IL-1α and IL-1β [48].

## Conclusions

We demonstrated that the systematic administration of aspirin and allogeneic ASCs attenuated bone loss more effectively than either one alone in OVX rats. Also, aspirin plays a key part in bone regeneration owing to its anti-inflammatory and chemotactic abilities. The results of our study will shed more light on the potential use of aspirin and allogeneic ASCs in the prevention/treatment of osteoporosis.

## Abbreviations

ALP: alkaline phosphatase
ASP: Aspirin
ASCs: Adipose-derived stromal cells
BFR: Bone formation rate
BM: Bone marrow
BMMSCs: Bone marrow-derived mesenchymal stem cells
BMSCs: Bone marrow stromal cells
CM: Conditioned medium
ELISA: Enzyme-linked immunosorbent assay
IFN: interferon
MSCs: Mesenchymal stem cells
P1NP: Procollagen 1 N-terminal peptide
RANKL: Nuclear factor-kappa B ligand
TNF: tumor necrosis factor
TRAP: Tartrate-resistant acid phosphatase
ZOL: zoledronate
